# Dialogic Learning Environments That Enhance Instrumental Learning and Inclusion of Students With Special Needs in Secondary Education

**DOI:** 10.3389/fpsyg.2021.662650

**Published:** 2021-06-16

**Authors:** Diego Navarro-Mateu, Teresa Gómez-Domínguez, María Padrós Cuxart, Esther Roca-Campos

**Affiliations:** ^1^Department of Inclusive Education, Community Development and Occupational Sciences, Catholic University of Valencia, Valencia, Spain; ^2^Department of Didactics and Educational Organization, University of Barcelona, Barcelona, Spain; ^3^Department of Comparative Education and Education History, University of Valencia, Valencia, Spain

**Keywords:** students with special needs, inclusive classrooms, special educational needs, secondary schools, inclusive education, instrumental learning, interaction, dialogue

## Abstract

Across Europe, the enrolment of students with special educational needs in regular classrooms is increasing, although it does not always mean access to high quality educational experience. In this context, inclusive education has been enhanced in most educational systems, but its successful implementation is still limited and has become a challenge in most countries, and specially in secondary education, when segregation due to learning achievement is more frequent. Educational practices that take into account the potential of promoting learning interactions within heterogeneous groups of students have already demonstrated contributing to educational inclusion of students with special needs. In this study we analyse the case of a secondary education school located in Valencian Community (Spain), which educates students with special needs along with their typically developing peers and is characterized by its inclusive ethos. The analysis focuses on three educational strategies implemented in the school and their impact on educational improvement and inclusion of the students with special needs: (1) co-teaching, (2) interactive groups, (3) dialogic literary gatherings. Qualitative data were obtained from communicative focus groups with teachers, communicative life stories with students and relatives, communicative observations of the three educational strategies and documentary analysis. The findings show significant increase in the students' instrumental learning, as well as an improvement in these students' overall inclusion in the school.

## Introduction

Currently there is a strong interest in addressing the inclusion of people with disabilities from international institutions and organizations. A clear example is the *World Disability Report* produced jointly by the World Health Organization and the World Bank (World Health Organization, 2011). This report formulates policies to make the implementation of the content of the United Nations Convention on the Rights of Persons with Disabilities a reality.

In this regard, the report notes the high rate of difference in school attendance between students with and without disabilities, both in primary and secondary schools, with emphasis on the latter. The report states the need to “adopt more learner-centered approaches with changes in curricula, teaching methods and materials, and assessment and examination systems” (World Health Organization, 2011, p. 15).

In line with the growing importance of the inclusion of people with disabilities, inclusive education is part of the fourth United Nations Sustainable Development Goal (SDG4), “Ensure inclusive and equitable quality education and promote lifelong learning opportunities for all.” Furthermore, the European Strategy for the Rights of Persons with Disabilities 2021–2030 (European Commission, 2021) clearly states that education centers must provide an inclusive approach to ensure the right of all persons with disabilities to participate in all educational levels and forms on an equal basis with others. The Strategy also acknowledges persisting gaps in educational outcomes between learners with and without disabilities, and a lack of research on the conditions necessary for learners with disabilities to succeed.

This clear normative engagement with the rights of persons with disabilities has contributed to the increasing number of students with special educational needs accessing primary and secondary mainstream schools (Eckes and Ochoa, 2005; Konur, 2006). However, the challenge of including students with special needs in mainstream schools remains. In Spain, where the study of this paper has been conducted, the report by the UN Committee on the Rights of Persons with Disabilities (United Nations, 2017) concluded that the initiatives and reforms toward inclusive education have not changed in deep the characteristics of the education system, which maintains violations of the right to inclusive and quality education mainly linked to the structural exclusion and segregation of persons with disabilities from the general education system on the basis of disability.

Moreover, the rates of students with special needs (SEN) in mainstream secondary schools are lower than in primary schools, despite the gap between the percentage of students with SEN in mainstream primary and secondary schools has reduced in recent years (Buchner et al., 2021). Mastropieri and Scruggs (2001) remarked, two decades ago, the complexities of inclusion in secondary education, ranging from academic complexity, pace of instruction and teacher attitudes. Worrell (2008) identified seven barriers that hinder the implementation of inclusive practices in secondary education: negative teacher perspectives; lack of specific knowledge; poor collaboration skills; lack of administration support; limited instructional repertoire; inappropriate assessment procedures; conflict between scheduling and time management. In the same vein, Verdugo and Rodríguez (2010) found specific difficulties for implementing inclusive education in Secondary Education, which are related to social interaction and the attitudes of classmates and professionals. Clark-Howard (2019) notes as specific barriers to implement inclusive education in secondary education the school culture, as well as standards pressure. The literature review by De Vroey et al. (2016) points out weak parental involvement and difficulties related to the curriculum and assessment, among others, as challenges to address, but also emphasize particular strengths of secondary education for the inclusion of students with disabilities (for instance, the active role of the students and peer relationships as a resource).

A comprehensive analysis of existing comparative studies from the 1990s on interventions for children with intellectual disability in mainstream or segregated settings already showed improved performance in academic achievement and social competence for those students learning in general education settings (Freeman and Alkin, 2000). However, research has also progressively shown that the participation of students in the mainstream classrooms does not in itself lead to the desired benefits (Comité Español de Representantes de Personas con Discapacidad, 2010; Florian and Black-Hawkins, 2011; Suriá, 2012; Lindsay and Edwards, 2013). In Spain, the Ombudsman for Children (Defensor del Menor en la Comunidad de Madrid, 2005) stated that a large number of secondary school students consider that their peers with disabilities are discriminated against in the classroom.

To revert the difficulties in the implementation of inclusive education and improve not only the percentage of students placed in mainstream education but also their actual academic and social attainments, research evidence points out the need of giving the opportunity for students with special needs to participate in activities together with their peers without special needs (Florian and Black-Hawkins, 2011). Teaching arrangements that improve the relationships that students with and without special needs have among them are important for improving the acceptance of students with special needs and their self-concept (Mpofu, 2003; Pijl and Frostad, 2010). Studies such as Carter et al. (2017) and Schmidt and Stichter (2012) in secondary education, show that peer support arrangements increase social interaction and academic engagement for adolescents with severe intellectual disability. In the same vein, Rillotta and Nettelbeck (2007) point out that for students without disabilities to interact with their peers, there must be a supportive environment that facilitates their interaction. In the last years, there have been advances in research exploring the strategies for implementing dialogic teaching and learning approaches in inclusive educational settings (Fernandez-Villardon et al., 2020).

This claim of expanding interactions in mainstream classrooms with students with special needs is aligned, in fact, with the growing recognition of the exceptional value of dialogue in any learning process (Mercer and Dawes, 2014; Resnick et al., 2015). Building on Vygotsky contributions (Vygotsky, 1978), current educational psychology places social interaction at the core of the learning processes (Mercer and Howe, 2012). Among the different theoretical approaches that delve into specific aspects of dialogue in the classroom, Flecha (2000) has developed the theory of dialogic learning based on seven principles (egalitarian dialogue, cultural intelligence, transformation, an instrumental dimension, the creation of meaning, solidarity, and equality of differences). Furthermore, the INCLUD-ED research project led by Flecha provided evidence on a set of successful educational actions that achieve both raising academic outcomes and social and personal development in very diverse contexts where they are applied. These actions are based on dialogue and participation of the community (Flecha, 2015).

One of these actions is Interactive Groups. It consists of organizing classroom in small and heterogeneous groups of students and place one adult in each group to facilitate the helping relationships between the students in the group so that they work together on the assigned task (Zubiri-Esnaola et al., 2020). Adults may preferably be not only teachers but also people from the community such as relatives or neighbors of the school, and other volunteers, such as university students. The adults and the activity to be developed rotate through the different small groups, so that in a typical 1-h session, each student has interacted intensively with his or her small group and with 3 or 4 adults.

Another of the successful educational actions identified by the INCLUD-ED project are the Dialogic Literary Gatherings in which participants dialogue around a piece of literature that they have previously agreed on and read in their own (Lopez de Aguileta, 2019). Importantly, the books are one of the works of the best universal literature, such as Shakespeare's Rome and Juliet, Cervantes' Quixote, or Homero's Odissey. Each book takes a set of DLG sessions, and the dialogue is initiated on the basis of the fragments that the participants have chosen to share with their peers. The aim is not to evaluate or correct the interpretations made but to share and deepen the reading.

There is extensive evidence on the social impact of dialogic gatherings and interactive groups in the educational and emotional improvement of typically developing children (De Mello, 2012; Flecha, 2015). In the case of children with disabilities and other special needs, research is increasingly showing that in interactive groups or dialogic gatherings, the learning of children with special needs is increased, not only in the academic domain in subjects such as mathematics, reading, writing, among others (Díez-Palomar and Cabré, 2015; Molina Roldán, 2015) but also in prosocial behavior (García-Carrión et al., 2020b). However, the analysis has been focused on contexts of preschool and primary education and special education centers (Duque et al., 2020). In contrast, the study of the impact of these actions on children with special needs in secondary education mainstream schools is almost non-existent.

Co-teaching has been implemented by many schools as a mean to respond to the challenges of having students with and without special needs in the same classroom. Usually consists on one general teacher paired with one special teacher who, in many cases has a subordinate role as an assistant. The existing studies have demonstrated the benefits of the strategy, even though there is a lack of evidence on the comparative effectiveness across different models and strategies of doing it (Iacono et al., 2021). Among other difficulties, it has been observed that the pairing of teachers often does not lead to increased peer interactions between students in the classroom (Scruggs et al., 2007).

In this framework, this contribution analyzes the case of a secondary education center located in Valencian Community (Spain) that serves typically developing children along with students with special educational needs (including developmental disabilities and learning disabilities) and has implemented Interactive Groups, Dialogic Literary Gatherings and Co-Teaching progressively since 2014. Our study is framed within the research project INTER-ACT, “Interactive Learning Environments for the Inclusion of Students With and Without Disabilities” funded by the Spanish National Programme for Research (2018–2021), which analyses successful educational actions and their impact on participation, on the cognitive dimension (instrumental learning and cognitive development) and on the socio-emotional dimension (social cohesion and emotional and affective development) of students. The case that we present is one of the success stories selected in the first phase of the INTER-ACT project.

As a result of the case study, we describe the process of transformation of a secondary school from segregated to inclusive environments, provide evidence on the dynamics generated in the classroom, and identify improvements in the learning, development and relationships of children with special needs (focusing on those with developmental disabilities). These findings contribute to fill in a gap in the current research on what works in inclusive education, in particular, how to successfully implement dialogic learning environments in secondary schools.

## Materials and Methods

There is agreement in the scientific literature that measuring the degree of inclusion in schools and its impact on children with special needs requires the analysis of success stories that are sustainable over time, as well as the integration of the diverse voices that participate in the community (Frederickson et al., 2007; Carpenter and McConkey, 2012; Carrington et al., 2017). Therefore, this study analyzes in depth the case of a secondary school that started a transformation process toward inclusive education more than 5 years ago. The study was developed following a communicative methodology, which allows us to analyze and understand in depth those relationships that are established in the school with respect to students with special needs. One of the essential principles of the communicative approach is the inclusion of the voices of all the affected by research, in particular those who have traditionally been excluded from the creation of scientific knowledge (Redondo-Sama et al., 2020). In recent years the inclusion of the voices of children with special needs in research has become very important with the aim of transforming the processes of discrimination and submission to which they have traditionally been exposed. The communicative methodology incorporates their voices in equal dialogue in all phases of the research. This dialogue has allowed researchers and end users to interpret social reality in a dialogical way, to generate knowledge aimed at further transforming inequalities and to understand the feelings and desires of their lives.

### The Case Study: Sorolla Secondary School

The Sorolla Secondary School (pseudonym), is a state school, located in Valencian Community, Spain. The school had an approach based on the segregation and individualized attention of children with special needs until 2014, when it began a whole school transformation process becoming a Learning Community, after being chosen by 99% of the families, 99% of the students and 70% of the teachers. Learning Communities is a whole school intervention aimed to improve learning and social cohesion, through the dialogic participation of all the community and the implementation of successful educational actions (Gatt et al., 2011). In the 2014–15 school year, the Sorolla School initiated the inclusion of children with special needs through the implementation of Interactive Groups (IG), Dialogic Literary Gatherings (DLG), and Co-Teaching (CoT). Today all students with special needs are served in classrooms with their typically developing peers. This center has been valued by Valencian Government as a successful center in the creation of mixed environments of educational inclusion through the pilot study carried out in 2019 in preschool, primary and secondary education centers (Generalitat Valenciana, 2020).

At the moment of the study, Sorolla Secondary School had 1,053 students of 26 different nationalities. It provides compulsory secondary education (357 students) and high school (134 students), middle and higher vocational training (208 students) and basic qualification training programs (9 students). The school also has a Specific Unit of Special Education that attends to 8 students with autism. The teaching staff consists of 116 teachers, 2 counselors, 3 special education teachers, 1 speech therapist, and 2 educators. This research focuses on compulsory secondary education, where 15% of the total students are adolescents with special needs (developmental disabilities, learning disabilities, and other special needs such as mental disorders). Specifically, it has evaluated the impact that dialogic learning environments are having on children with developmental disabilities (i.e., autism, intellectual disability, hyperactivity, etc.), who make up 9.5% of the students. Other students with disabilities such as dyscalculia, dysgraphia, or dyslexia are not analyzed here. Table 1 shows the students with special needs participants in the study and the associated educational needs.

**Table 1:** Children with developmental disabilities participants in the research de 2018 a 2021.

**Developmental disabilities**	
Hyperactivity disorder	10
Moebius syndrome	1
Motor disability	1
Visual disability	1
Intellectual disability	7
Autism spectrum disorder	11
Turner syndrome	1

Currently, the school is developing several actions that involve community participation and contribute to create an inclusive environment for all students, including the dialogic model of conflict prevention and resolution (Serradell et al., 2020), dialogic training for families and teachers, mixed committees, and a tutored library. This paper analyses the main actions that the school has introduced in the schedule to guarantee the inclusion of students with special needs, namely Interactive Groups (IG), Dialogic Literary Gatherings (DLG), and Co-Teaching (CoT).

### Data Collection and Analysis

Data collection took place during the 2019–20 and 2020–21 school years (see Table 2). Each of the instruments is described below.

**Table 2:** Data collection instruments.

Communicative focus groups (CFGs)	2 with management team and head of counseling department (total 3 participants each CFG) 1 with 3 teachers and 4 members of the counseling department (total 7 participants) 1 with students with (3) and without (4) special needs (total 7 participants)
Life stories (LSs)	2 mothers of students with special needs 1 student with special needs 1 student without special needs
Communicative observations (CO)	Students with special needs
Documentary research (DR)	Reports of the school not available in the public domain

#### Communicative Focus Groups

We held four communicative focus groups with different participant profiles. On the one hand, two communicative focus groups were carried out with the management team and the director of the counseling department, who were able to provide an overview of the work with students with special needs in the center and specifically from the educational actions studied. Both groups were made up of the director, the vice-director and the educational advisor of the center. The first communicative focus group was carried out at the beginning of the investigation and information on the processes of progressive incorporation of the IGs, DLG, and CoT was jointly analyzed. The second communicative focus group was carried out at the end of the research to share and validate the results on the impact of these learning contexts on children with special needs.

On the other hand, one communicative focus group was held with teachers and members of the educational counseling department that serves students with special needs in their classrooms. In this meeting, they discussed the processes of transformation of teaching through contexts of dialogic interaction with children with special needs and typically developing children. Likewise, the teachers explained the impact observed in the cognitive and social development of the students with special needs. The communicative focus group was made up of a member of the management team, 2 special education teachers, the speech therapist and 3 teachers of various subjects.

Finally, one communicative focus group was carried out with students with special needs (3) and with typical development (4) who have participated in the IG, DLG, and CoT actions for at least 2 years. All of them are students of the second year of compulsory secondary education (13–14 years old). The adolescents discussed how GI, DLG, and CoT were developed, to what extent and why it facilitated the learning of children with special needs and what kind of relationships were established between the students through these actions.

#### Comunicative Life Stories

We developed four communicative life stories. The first one with one student with special needs about his experience after 2 years of participating in dialogic interaction contexts. The second was to one typically developing adolescent about the impact of his friendship on the development and learning of another adolescent with special needs. The third and fourth were held with two mothers of other students with special educational needs. The objective of conducting these life stories over a short period of time was to dialogically reconstruct the reality lived by the students with special needs, expanding the understanding of their experiences, thoughts and feelings. The narration from different people who share a daily life with these students allowed a multidimensional understanding of the impact of these learning contexts on the development of children with special needs.

#### Communicative Observations

We made four communicative observations of IG (2), DLG (1), and CoT (1) involving children with special needs. In these observations, the type and quality of interactions that took place in the classrooms between students with and without special needs, the teacher and other adult participants were analyzed. The categories used in the analysis were: the participation of the student with special needs in the learning activities, helping relationship, friendship relationships (looks, laughs, comments…), the degree of adult intervention in the interactions, adaptive behavior.

#### Documentary Research

We carried out a documentary research of internal reports and memoranda of the educational center. This documentary research made it possible to analyze two issues. On the one hand, to know in depth the process of transformation of teaching contexts in groups of segregation, to contexts of inclusion in dialogic interaction. This research process allowed the researchers and participants to reconstruct the processes of progressive incorporation of the IG, DLG, and CoT and to make it easier for other schools to reproduce them (Grant, 2020). On the other hand, we had access to documentation on the evaluation of the learning of the students with special needs corresponding to the school years 2018–19 and 2019–20. This allowed us to assess the impact of 4 years of progressive application of the IG, DLG, and CoT on the learning and curricular planning of students with special needs.

Our position as researchers was to understand the features, positive impacts and difficulties in the implementation of dialogic learning environments in secondary education, with the aim of providing evidence to be used in the improvement of inclusive education. The management team of the school was very interested in the research project in which the study is framed and was aware of its orientation toward social impact and. Besides, one two of the authors have had previous collaborations with the School. This positioning facilitated the access to the fieldwork and a smooth communication with the school during the whole process.

### Data Analysis

Following the postulates of the communicative methodology, the dimensions of analysis have been, on the one hand, the exclusionary dimension, that is, those difficulties that occur in these learning contexts based on communicative interaction; on the other hand, the transformative dimension, which identifies those elements that make it possible to overcome existing inequalities in the care of children with special needs. These two dimensions are transversal to the categories of analysis. The categories observed are, firstly, the process of incorporation and transformation of a secondary school from environments based on segregation to environments based on dialogic interaction and inclusion. Secondly, what are the main improvements identified so far from the different agents involved in these contexts. These categories have been created in a deductive way taking as a reference the analysis of the scientific literature. This has made it possible to identify as categories of analysis the main challenges that schools currently face in order to achieve a fully-fledged education for special needs' students in compulsory secondary education (see Table 3).

**Table 3:** Description of analysis categories.

Learning outcomes	What results do students with special needs achieve and what is their academic progression.
Quality of learning interactions	To what extent do students with special needs participate in learning activities and content with typically developing students.
Quality of social relations	If there is a relationship of help, friendship, solidarity with the students with special needs.
Attitudes and beliefs toward special needs' students	To what extent there is a transformation in the outlook and expectations toward students with special needs.
Adaptive behavior and self-regulation	Participation of students with special needs in classroom routines with typically developing students.
Impact on the teaching role	There is a change in teaching staff, organization, and beliefs toward the inclusion of students with special needs.

Each interview was analyzed according to these categories and dimensions, and researchers reached intercode agreement through crosschecking their codings, for greater credibility. Besides, the diversity of techniques and informant profiles has facilitated the triangulation of information and, as part of the communicative approach of the study, researchers maintained an ongoing dialogue with participants in regards to the interpretations of the data meanings. This member checking was complemented with a final communicative discussion group with the management team and the counseling department, in which the findings were fully discussed. We did not undertake an external audit besides the evaluation of the project design prior to be funded under the national competitive call for research projects.

As a single case study, the transferability of the findings must be considered cautiously and take into account a wider body of research.

### Ethics

All participants (teachers, families, and students) have agreed to provide the members of the research team with relevant information to achieve the research objectives. The different participants have been informed about the purpose of the research, the participation has been voluntary, as well as the confidential use of the collected data, which will be exclusively used for the purposes of the research. They were provided with written informed consent. Names appearing in the text are pseudonyms. The set of ethical procedures established by the European Commission, 2013 for EU research, the data protection directive 95/46/EC and the Charter of Fundamental Rights of the European Union (2000/C364/01) have been followed and complied with. The study “*Dialogic learning environments that enhance instrumental learning and inclusion of students with special needs in secondary education”* was fully approved by the Ethics Board of the Community of Researchers on Excellence for All (CREA)^1^.

## Findings

In what follows, we present the findings obtained through the case study of Sorrolla secondary school. First, we reconstruct the story of the progressive development of dialogic learning environments in the school. We have considered mainly the perspective of the management team and the guidance department. Later, and based on the voice of students, families, and teachers, we describe the features of interactions promoted in the analyzed dialogic learning environments and the impact of these environments on the learning outcomes, social development, and relationships of children with special needs.

### The Process of Change Toward Inclusive Dialogic Learning Environments

Prior to its transformation through dialogic interaction contexts, Sorolla Secondary School approached the educational intervention with children with special needs through segregation practices. Some of these practices, widespread in the Spanish educational context, were the PAE program (Programa de Acompañamiento Escolar—School Accompaniment Programme) or the specific attention to children with special needs outside their reference classroom, in homogeneous groups or individually.

During the academic year 2014–15, successful educational actions (Flecha, 2015) were introduced in the school, such as the Interactive Groups (IG) and the Dialogic Literary Gatherings (DLG). This was done on a voluntary basis by part of the teaching staff. One of the measures adopted was the inclusion of students with special needs in the classroom with other students when these actions were being implemented. The special education and speech therapy teachers started to devote some hours of their teaching within the classrooms in which students with special needs were now placed, instead of doing solely separated interventions. Later, during the 2015–16 school year, the application of IG and DLG was generalized in the instrumental areas (Spanish, Valencian, English, and Mathematics) of first and second secondary education grades. One year later, in the 2016–17 school year, the IG and DLG were extended to all secondary education courses. During the 2017–18 school year, two other actions were introduced on a pilot basis: co-teaching and heterogeneous splitting.

The heterogeneous splitting consisted in reducing the ratio of students dividing the groups while maintaining diversity in each group and one teacher per group. Co-teaching meant that two teachers (at least) intervened in the same classroom, maintaining the size, and diversity of the groups. The management team explains that they obtained better results in those classrooms that had been carried out the pilot in co-teaching, than in those that had been carried out heterogeneous split:

Although during one academic year, 2017–18, the will of the teaching staff was respected and heterogeneous group splits were carried out, reducing the ratio of students by half, this measure in itself did not produce an improvement in results. Those groups in which two or more adults were introduced into the classroom, making the interactions more dynamic, increased their results, but not those in which the ratios were reduced. [CFG_ coordination team]

Following these results, the school decided in 2018–19 to establish co-teaching as a regular measure in the first and second grade of secondary education at the school, when it is not possible to provide IG. This way, the school achieved that students were attended within the classroom more than 60% of the schedule by two or more teachers and adults from the community. In that academic year, the school also achieved that children with special needs were included in their classroom during 95% of the school time. The IG sessions were doubled from biweekly to weekly, prioritizing this measure in the first and second grades. This was made possible by the participation of 122 volunteers from the community (mainly parents and other relatives of students). Children with special needs worked on the same content as their peers and an individualized plan was applied to them on the evaluation criteria.

Both the management team and all the teachers interviewed at the Sorolla school agree that this process of inclusion of children with special needs could not have worked without the environments of dialogic interaction. The teachers of the counseling department explained in more detail that the process of incorporating children with special needs has not been automatic but a progressive change at different levels. The speech therapist insists on the fact that the process entailed specific adjustments to each adolescent with special needs.

Speech therapist: Yes, of course, adjusted to each student. It has nothing to do with these children when they arrived in first grade than now that they are already in 4th grade. At first, they were very nervous, restless, and it has been a very important process of self-control of their own behavior. It was a process of recognizing the physiological responses that these situations created for them (sweat, nervousness, mannerisms, etc.) and all of this was worked on through self-instruction and respecting their freedom and needs if they had to get up and leave. It has been a long and laborious process since they came very protected from primary education, from segregated environments. They came very well-worked on habits, but all that is autonomy, they did not have. [CFG_Speech therapist]

But the process has not entailed only adjustments for individual students with special educational needs. Another teacher emphasized the effort it has meant for all the involved.

This has been a collective long-distance race. The teenagers, the teachers, the families… all together… this is not like magic, it is the joint and constant work of all these actions that make it possible… now we are telling what started in 2014 [CFG_member of head teacher team]

This same reflection, including families as an agent at the level of the pupils and teachers, reflects the cultural change and the change in human relations that has culminated in this process.While the process has been a progressive change both at the individual level for the students and at the school level, the teachers who have joined the school when the changes had already been made find a very different approach than in other schools they know. This is described by an English teacher in his first year:

It is my first year at the center and I quickly detected the relationships of solidarity and inclusion, compared to other centers. It is the students themselves who take care of and help manage the children with special needs, so that they can properly follow the organization of the classroom. For me it has been a very positive experience, which scared me at first because there were other adults in the classroom… but now I am delighted… [CFG_English Teacher]

Again, one of the members of the management team identified himself with this feeling of fear in the presence of volunteers in the classroom, a fear that disappeared in the course of the implementation process.

I understand you because at first, it's scary because you've never worked like this, with more people in the classroom…, and when you start and see the results, it's just the opposite, you see that it's logical, common sense to work like this, for children with special needs, but also for everyone. [CFG_ Teacher 1]

In fact, the full participation of children with special needs in mainstream classrooms led to a profound change in the role of special education teachers themselves. In the next excerpt from a Communicative Focus Group, two members of the counseling department illustrate this:

Special education teacher 2: My experience as a special education teacher until I came here was like many in a secondary school. You have your classroom, where you take the children with special needs, separated into small groups… and of course, from that point on we are all stigmatized, the children and I, because I no longer relate to the rest of the teaching staff practically, except for specific moments and the children, because the same thing. All of a sudden you arrive here and you no longer have classrooms, all of them are your classrooms… I… sincerely believe that it is very important not to stigmatize the students, but also, as a resource, I am very optimized.Counselor: we really wouldn't know, we don't want to work in segregated contexts anymore.

#### Multiplying and Diversifying Interactions of Children With Special Needs

In all the interviews and discussion groups conducted, students, families and teachers emphasized the importance and richness of interactions promoted in the three strategies analyzed. For instance, one of the students with special needs interviewed defined the school's way of doing as promoting interactions that help him to learn.

The best way to learn is with the teachers, with the classmates and with other people… and this is what the institute does… and I don't know if it's the best answer, but it's what I think, what helps me learn [LS_student with special needs 1]

In the framework of a school that multiplies interactions, DLG and IG are the actions that stand out the most. As it has been already explained, IGs consist of small groups working together with the mediation of an adult in each group, who has the role on promoting interactions rather than providing individualized support within the small group. A student with special needs emphasized that the IGs allow him to follow the learning path of the class:

I think that's how I learn because in the group we help each other when someone loses the thread of the class. This is good for me because if not, you don't learn anything, I say this with respect. When you are at IG you have someone to explain the exercises to you and you don't do it alone. [LS_student with special needs 1]

The interactions within the IG involve a repetition and diversification of messages and task instructions among the group, through which children with typical development do a communicative scaffolding with children with special needs. We introduce here a dialogue between five students where they explain these interactions:

Student 1: The difference between IG and normal groups, well, we go together all at the same time, while as normal, we go separately, and it is better to go together, we talk, and we speed up.

Student 2: Yes, this way time passes more quickly, and we concentrate more.

Student 3: Well, because I think that, if the teacher explains something and we understand him, well, but, if we don't understand him, there is always someone in the group who can explain to the student what the teacher has just explained in other ways, and maybe he understands it better.

Student with special needs 2: There was a day when I didn't know anything about math and my friend, who knows a lot about math, helped me and I understood, and I got to pass the exam.

Student 1: We help him to repeat it again, to see if it stays in his head.

Student 4: What we do is, if a kid doesn't understand it, try to explain it to him more slowly and in a way that the whole group can understand, and see if that way he gets it.

Researcher: And has it ever happened to you that you explain it to him and he doesn't understand you?

Several students: Yes.

Student 1: Then we repeat it to him, let's see if this way…

Student 5: And sometimes we try to repeat it to him, in other words, so that he can understand it better.

Student 4: I think that before, they were ashamed to say it [student with special needs], once we did the group thing, he asked for help and then there was no more shame.

Researcher: And do they also ask for help outside the interactive groups? Or do you only help each other in interactive groups

Several students: in everything!

Student 4: Yes, but thanks to the groups, I think.

One student told us about her autistic friend: “with these actions, Alberto has been able to communicate more and with more people, not only with me” [LS_student without special needs 1]. In the same line, a student explains from her personal experience, how interactive groups force interaction that in turn create more stable relationships:

Excluding people is not right either. In IG we help each other, if they don't understand something, they can ask about it and if they are excluded maybe they can't understand so well when a teacher explains or someone else. And also, that they have to interact with others, because if they are excluded, they don't relate to the other children. Yes, there may be people who have a little more difficulty in relating and, in the Interactive Groups, as you have to do because it is part of the work, then it helps you interacting with people. Then you already create relationships [CFG_Student 3]

Nevertheless, some students with special needs find more difficulties when working in interaction with their peers and thus not all of them express the same satisfaction with the enriched environment of dialogic interaction. Interestingly, the mother of one of those students explained her own positive perception of the interactive groups:

Mother: My daughter [a student with Asperger's syndrome] complains a lot that she memorizes a lot here and she likes it to be explained, to be reflected upon. She doesn't like to read. She has a hard time with school work because it doesn't pique her interest. Interactive Groups, for example, she tolerates them, but doesn't like, because there is too much interaction for her. [LS_Family_student with special needs 2]

Researcher: And do you think that these spaces of controlled interaction are good for her, even if she doesn't like them?

Mother: It's good, no, the following! Because I think it's important not to stop interacting with her and to always be attentive to that interaction that little by little manages to awaken interest, of course, respecting her space, whatever she needs, but without stopping trying. She has improved a lot in all subjects. If we were not continuously pushing Azucena…, I don't know if we would be talking now… because she was proposed for the specific center. Before she didn't interact with anyone, she didn't sit with anyone, she didn't talk to anyone, in the previous school she couldn't go out to the blackboard to say anything, to have someone helping her to put her jacket on was a trauma. I think that's the key, to continue interacting and not stop doing it… [LS_Family_student with special needs 2].

In the case of the DLGs, both students and teachers stress the topics that emerge from the classic literature works as a key factor for increasing their motivation and participation in dialogues.

I like the gatherings a lot because of the topics they deal with. About love I am very interested, I like very much that when someone is in love with someone, how happy love is. I'm also interested in death, because that's how books talk about some families that have died, and it's sad, but I like to talk about it because, although it makes me sad, people have died in my family too, and of course, these are subjects that interest me to talk about [LS_student with special needs 1].

Also, for example, dealing with important topics that in another segregated environment would not be dealt with, sharing experiences with others is very enriching. In the other type of teaching [segregated] this does not happen and there is a lack of motivation, in these actions the motivation increases. I talk with other students from other schools who don't work like this…, and that the adolescents with special needs only share the patios and some sessions, I think that the stereotypes, phobias, the language, the communicative capacity…, come on… I think it would be almost the same as how they arrived [CFG_Speech therapist].

Moreover, the methodology of DLGs favor the participation of children with special needs in a structured and prepared way.

The gatherings have encouraged respect for these students, who are valued because they are amazed at their interventions, even when sometimes very affected students have made interventions that have nothing to do with the text [CFG_Head teacher team].I would even say that this respect is easily observed because when they speak it is more silent, because they are aware of the difficulty, they may have in expressing their ideas… and this solidarity is created… [CFG_education special teacher 1]

The teachers agree to express that the dialogic literary gatherings offer security to children with special needs to participate in learning. In the classroom observations, we were able to see that interventions from students without special needs served as modeling interventions. This has resulted in children with special needs now participating in the discussions with autonomy and interest. For example, one of the teachers explained the case of one of her students:

My student with educational special needs is the best at preparing the discussion because she loves them, and they have allowed her to pick up a reading habit that her mother is excited about, she says, “is that she wants to read more and more books since she's in secondary school.” In the gatherings, the student feels confident and very motivated to participate. [CFG_Teacher 2]

One of the teachers of the language area summarized the feeling she had about the dialogic environment context that has been created for students with special needs as a continuous stimulation, in which interactions have been multiplied and diversified:

Without these actions these students would be much more isolated and at a communicative level, they would have much less capacity. If you wish, they are always in communicative interaction, they always have people around them who are communicating among themselves, with others, all those interactions that surround them already give them a lot [CFG_teacher 4]

In turn, the interviewees consider that this environment has promoted individual changes in terms of learning, but also on how children with special needs are valued by their peers.

Reflecting all the impact of these actions on the students with autism that I attend, after 4 years I see that it has been very positive, at a curricular level, since they have seen their skills increase. At the level of relationships, because they have managed to integrate into a totally normalized context and at the same time, and something very important is that it has transformed the vision that the other students had of them (to be part of the class whatsapp, of their meetings, etc.) and even, that of the teachers, their vision has changed… [CFG_ Speech therapist]

In the following sub-sections, we focus on these impacts on both the socio-emotional dimension and the academic achievements.

### Impact of Dialogic Interaction Contexts on Students With Special Needs' Socio-Emotional Dimension

Our data suggest that the interactions promoted in the dialogic learning environments that we have analyzed have relevant impacts on children with special needs' emotional and affective development, participation and relationship with others. Interviewees highlight the feelings of self-esteem and self-concept, improving their motivation for learning activities. One of the center's special education teachers explained it this way:

I think that emotionally they feel more balanced because they see that they are doing the same activities as their peers and are always trying to excel. They see that it is difficult for them, that they are not like the others, but they try, because they want to get where the others are going…, to do what they do and it is very fulfilling for them to feel this way, they are very motivated. [CFG_ special education teacher 1]

In the same vein, the Speech therapist has noticed changes even on their physical appearance, that hat are attributable to the fact that they are actively and regularly participating with their peers:

They have changed even on a physical level. They arrived with very childish behaviors and not at all adolescent and of course, now they take care of their image, their self-esteem… they have learned to understand the double meaning of language… “locked up” in my classroom [specific communication and language classroom] they would have ended up isolated, since they arrived with enormous stereotypes, and all that has been decreasing, decreasing… since they are in the ordinary context, they realize that they want to look like others, share interests with others to be accepted and try to share with them, share their lives. I think many times, what would have become of these young people if they had not been lucky enough to find an institute that promoted these actions! [CFG_ Speech therapist].

Also a mother of a student with special needs highlights with enthusiasm the feeling of being “one more” (both for her son and herself) generated by participating in all the activities. This inclusion has allowed his son to regain the excitement and enthusiasm in his life and in his learning:

Above all I want to talk about how important it is for my son to be in this center, how happy it makes him, how happy he is. He feels like one more, he participates in all the activities. He needs less and less help, his material is less and less adapted… my son was in a special education center, my son and I do know what we like or don't like, and we know how good it is to be in a school like this that is inclusive. In the other school, the door was closed all the time… My son wants to feel like a person, one more in this society, and here they are showing us that he can be, he feels like one more, I also feel like one more mother. [LS_Family_student with special needs 1].

Besides teachers and relatives, the IGs are explained by the students without special needs as the action that allows them to create those relationships of friendship and solidarity with children with special needs:

Student 4: Well, when we work in a group, what you do is talk, and when you talk, you make more friends and get along better with them. We laugh together and, in the end, we make friends

Student 1: I don't remember if last year or this year I saw some kids in the 3rd grade of ESO, who are now in the 4th grade, getting along very badly. And then, I looked out the window of the door, and I saw them cheerful in the interactive groups and talking and all that. I don't know if I explain myself… I think that's why we work like this…

A finding that was not initially sought has been the improvement in the core handicaps of each of the associated disabilities. Special education teachers have highlighted that, for example, in students with autism and a communication and language disorder, there is improvement in social relationships, communication, and continued interaction with their non-disabled peers; or, one of the students with intellectual disability, who also had dyslalia, improved in text comprehension and motivation toward language learning.

Despite this very positive appraisal of the social relationships, a member of the Head teacher team, and it was contrasted with the rest of the teaching staff, highlighted a barrier that the center had yet to overcome. The teachers commented that, although in the school the friendship and good relations between the children with special needs and the other students can be observed, this had not yet been transferred to other spaces outside the school.

The motivation and commitment of the teaching staff, as well as the students, is understood when we analyze the sense that has been growing as the voices of students with special needs have been part of the life of the center, its decisions and the relationships that emerge.

At the beginning you arrive and say, “here they are not well” and now… I don't change this for anything… it is wonderful the relationships that are created and the sense that it gives you to see as adolescents that in other places would be marginalized, here we take them forward [CFG_special education teacher 2].

This way of working is educating everyone, students and teachers, what is lived here changes us all. The fact that the kids [students with special needs] work in this way, all together, doesn't mean that they don't adapt to their needs. If Azucena needs to leave the classroom, if Ivan doesn't want to leave for anything in the world, he doesn't leave. They have a voice to decide also, because they are all very different. This way of working makes them feel very welcome [LS_Family_student with special needs 2].

Indeed, these students now have more voice, but it is not only that we give them voice, it is that their peers are giving it to them and that is very important for them. For example, in the interactive group work, they are the ones who manage their voices, and they give themselves a voice and try to make them [students with special needs] have it [CFG_teacher 3].

#### Impact of Dialogic Interaction Contexts on Students With Special Needs' Learning Outcomes

It is not possible to make an analysis of the learning improvements of this student body from external evaluation tests because they are generally not implemented to children with special needs. However, the center has relevant data that we have been able to analyze corresponding to the academic years 2018–19, 2019–20, 2020–21.

On the promotion rate of students with special needs in 2018–19 and 2019–20 the data indicates that 100% of students with special needs graduated from compulsory secondary education without exceptional measures. This is a total of four students. Three of these students are currently in middle school without curricular adaptation. During the 2018–19 and 2019–20 school years, the percentage of children with special needs who are promoted to the next grade is 83 and 73%, respectively. The absenteeism rate for these students is 0%.

The Speech therapist who mentors students with communication and language disorders and autism comments on the improvements observed in the student body as follows:

I think this way of working helps them a lot, in the learning and also in the nuclear aspects of autism. Our students are learning more than before through these actions, and I have realized that the ordinary context is essential for them to learn more. You realize, for example, that it increases their vocabulary, the ability to better structure sentences, to use words more appropriate to each context, waiting times, tolerance to frustration, all these things I have seen that above all in the Interactive Groups and the Dialogic Literary Gatherings the impact has been very strong. And at the curricular level as well, since they came to the institute with their fourth or fifth grade book thinking that we would continue where they had left off in school, but no, here the expectation is different and we prepared the materials so that they work the same as the others… this has meant a spectacular jump in many of them in certain subjects, even matching the level of the others in some of them. And it is true that they still have their difficulties… but the changes are very big… [CFG_ Speech therapist]

In this sense, other relevant data observed is the reduction of significant curricular adaptation measures. In Spain, individual measures for curricular adaptation were widely introduced in the 1980s for children with special needs. When applying these measures, the teaching staff together with the guidance department establishes the learning objectives for the involved children based on their previous knowledge. This measure is applied to children with special needs who have a gap of 2 or more grades in the learning objectives with respect to their reference grade. In Sorolla school, according to the average of the last three academic years (2018–19, 2019–20, 2020–21), the index of children with special needs who present an extraordinary measure of significant curricular adaptation has been reduced from 72.22 to 50%. While in the 2018–19 school year, 13 students out of 18 had a significant measure of curricular adaptation, in the 2020–21 school year, 15 out of 30 children with special needs enrolled had one. The mother of one of the students explains the progress she has detected in her son in changing from curriculum adaptations to participating in these learning settings:

Before he copied two sentences and got tired… now he writes whole pages well and takes an exam. He's looking forward to doing well. What is most difficult for him is the theory because he can easily lose focus [mother_student with special needs 1]

In fact, the decrease of curriculum adaptations occurred at the same time as an increase of the enrolment of students with special education needs. In the 2018–19 school year there were 18 students with special needs enrolled (13 of them with a significant measure of curricular adaptation) and in the 2020–21 school year, the number of students with special education is 30 (15 of them with an adaptation).

The increase of students with disabilities may reflect a “magnet effect” among families who are looking for an inclusive school, regardless of whether they are eligible for this school according to their area of residence.

## Discussion

The findings that we have presented suggest that adolescents with special needs at Sorolla Secondary School benefit from dialogic learning environments with typically developing students, and are therefore consistent with learning theories that point out that interaction, dialogue, and small group work promote children's learning in general, and for students with special needs in particular.

Through IG, DLG, and CoT, students with special needs participate in the activities with their classmates and share the same learning contents. This way, the school reverses the frequent exclusion of students with special needs from culture, curriculum learning expectations, and decision-making in mainstream schools because of the deterministic beliefs in place (Florian and Black-Hawkins, 2011). The choice of the best literary creations of humanity in the case of DLG and the intensity of group work in IG entail high expectations for learning for any group of learners (Flecha, 2015), and thus even more for students with special needs. The qualitative data that we have obtained confirm these high expectations are perceived as very positive by the students with special needs, who in some cases highlight their engagement with the deep themes of classical literature. Moreover, teachers and peers do also change their perceptions about the interests and capabilities of those students. The research has revealed, in contrast to what some professionals of the Sorolla school previously considered, that these children with special needs are interested in topics such as love, friendship, death, and human conflicts. These findings shed light on opportunities for overcoming the perception of academic complexity as a barrier to inclusive practices in secondary school.

Beyond the learning content, what defines IG, DLG, and CoT is the multiplication of interactions in the classroom. The scientific literature examining peer-mediated interventions (Carter et al., 2017) has already demonstrated their benefits for enhancing the social interactions of students with disabilities and special needs. The existing reviews on peer-mediated interventions, however, tend to focus on specific activities in which some non-disabled students are especially prepared for providing support to students with disabilities. In contrast, in the IG, DLG, and the CoT that we have analyzed the adults have a very important role in promoting the maximum number of interactions by the maximum number of people (Flecha, 2000). These are therefore learning spaces where the interaction between equals is adult-mediated and progressively normalized in the dynamics of the classroom that opens up other possibilities to peer-mediated interventions in the context of secondary education.

Furthermore, adults are not necessarily and not solely teachers and specialized professionals but also students' relatives and other adults from the community. Previous studies have analyzed the value of volunteers in Interactive Groups (Valls and Kyriakides, 2013). The experience of the Sorolla School is consistent with these previous results, showing that volunteers with no specialized training may make positive contributions to promoting peer interactions in the classrooms. This finding has implications for current research on the roles and preparation of paraprofessionals who work with students with disabilities (Brock and Anderson, 2020) and the implementation of effective collaborations within inclusive educational settings.

Many adolescents with special educational needs, as a consequence of their affectation, have more difficulties to provide meaning to some social interactions. For example, some people with autism have difficulties in social relations because of their poor ability to interpret gestures and other actions with social meaning (Kandel, 2018). In line with previous research (Wehmeyer et al., 2003) teachers have clearly identified the improvement of adaptive behaviors in the classroom and school.As a result of the participation of children with special needs in IG, DLG, and CoT some of their non-socially adjusted behaviors, such as stereotypes or mannerisms, have decreased and they have developed more adaptive behavior. In the case of DLG, they have reduced the disconnection that they usually suffer from the human world around them, such as their desires, intentions, and beliefs (Bruner, 1997).

Finally, in line with literature on the changing role of special education teachers within the inclusive school framework (Durán and Giné, 2011) our study shows that the change of role at the organizational level (from teaching based on individual learning to teaching based on dialogic learning) has been accompanied by a transformation in the teachers interviewed at the individual level on two relevant aspects. On the one hand, on the expectation of learning toward children with special needs; on the other hand, on the importance of incorporating their voices in the life of the educational community. The stories of the teachers show that after their experience through the IG, DLG, and the CoT, their understanding of children with special needs has changed and that it has grown a shared desire for a better education for them.

The case of Sorolla School questions the model that has prevailed in Spain for children with special needs since the 1980s, marked by segregation and specific individual measures based on the concept of “prior knowledge” rather than on interactions that promote progression to higher levels of learning (Lopez de Aguileta and Soler-Gallart, 2021). Our study shows the feasibility of promoting approaches based on Vygotsky (1978) or Bruner (1997) contributions, which have been poorly transferred to educational practice with children with special needs in many countries.

We can conclude that IGs, DLG, and CoT in Sorolla School are increasing the opportunities to create learning environments closer to a fully inclusive learning situation for children with special needs. Our findings contribute to a research interest on the social impact of dialogic teaching and learning (García-Carrión et al., 2020a). This has clear implications for the professionals of the secondary education and also for the design of public policies. Educational centers like Sorolla Secondary School, and many others that exist worldwide, may inspire new educational realities.

## Limitations and Further Research

This study has some limitations. Firstly, we do not have the perspective of relatives and other adults who are volunteering in the school, and we have not included classroom observations that would allow to describe in detail the interactions that take place, such as the type of questions and comments that students with special needs ask and the frequency of these. Second, we do not have external evaluations to compare academic achievements before and after the implementation of dialogic learning environments. Third, and important, the findings are related to a single, particular case study. Despite other schools are implementing Co-Teaching, Interactive Groups and Dialogic Literary Gatherings, we have not compared the features of the implementation in groups with students with and without special needs, neither the impacts of this implementation. Therefore, the findings are highly relevant for the understanding of inclusive strategies in secondary education, but cannot be generalized. Finally, as the school has a relatively short experience in the implementation of dialogic learning environments, the sustainability, and the longer-term effects of the actions that we have analyzed here would require sustaining and updating data collection.

Further research should address these limitations and expand the analysis to other secondary schools. One of the issues that have a tremendous potential for the implementation of dialogic learning environments and requires more research is the role of relatives and other volunteers in the classroom and the modeling they can create to encourage peer interactions between students with and without special needs. A second topic of interest is to explore in more depth the effect the early incorporation of students with special needs into dialogic interaction environments has on the main difficulties associated with specific deficits. Finally, families and teachers consider a pending challenge to see to what extent the dialogues that arise in environments like DLG or IG can be extended to other spaces in the school, such as other classes, the playground, or activities outside school hours. Our research suggests that progress can be made in deep friendships growing between children with and without educational special needs in these environments, but it needs also further research efforts. In any of these future lines of research, the voices of students with special needs and their families, together with teachers and other actors involved, are of utmost importance. Ivan, Azuzena, and other students who shared their life stories with great communication effort and generosity did so because they want many other adolescents and families, whom they do not know, to grow up in hope that it is possible to expand their learning and development and surrounded by friendship and solidarity.

## Data Availability Statement

The raw data supporting the conclusions of this article will be made available by the authors, without undue reservation.

## Ethics Statement

The studies involving human participants were reviewed and approved by Ethics Board of the Community of Researchers on Excellence for All (CREA). Written informed consent to participate in this study was provided by the participants' legal guardian/next of kin.

## Author Contributions

MP and ER-C conceived the original idea with the support of DN-M and TG-D. DN-M and TG-D conducted the literature review. ER-C coordinated the data collection and transcribed and analyzed them. ER-C wrote a draft of the manuscript with the support of DN-M and TG-D. MP revised it and included corrections with the support of DN-M and TG-D. MP revised the final version of the manuscript.

## Conflict of Interest

The authors declare that the research was conducted in the absence of any commercial or financial relationships that could be construed as a potential conflict of interest.

## Footnotes

^1^The Ethics Board was composed of Dr. Marta Soler (president), who has expertise in the evaluation of projects from the European Framework Programme of Research of the European Union and of the European projects in the area of ethics; Dr. Teresa Sordé, who has expertise in the evaluation of projects from the European Framework Programme of Research and is a researcher in the area of Roma studies; Dr. Patricia Melgar, a founding member of the Catalan Platform Against Gender Violence and a researcher in the area of gender and gender violence; Dr. Sandra Racionero, a former secretary and member of the Ethics Board at Loyola University Andalusia (2016–2018) and a review panel member for COST action proposals in the area of health; Dr. Cristina Pulido, an expert in data protection policies and child protection in research and communication and a researcher in communication studies; and Dr. Esther Oliver, who has expertise in the evaluation of projects from the European Framework Programme of Research and is a researcher in the area of gender violence.
